# Gene Expression Profile in Primary Tumor Is Associated with Brain-Tropism of Metastasis from Lung Adenocarcinoma

**DOI:** 10.3390/ijms222413374

**Published:** 2021-12-13

**Authors:** Yen-Yu Lin, Yu-Chao Wang, Da-Wei Yeh, Chen-Yu Hung, Yi-Chen Yeh, Hsiang-Ling Ho, Hsiang-Chen Mon, Mei-Yu Chen, Yu-Chung Wu, Teh-Ying Chou

**Affiliations:** 1 Department of Pathology and Laboratory Medicine, Taipei Veterans General Hospital, Taipei 112201, Taiwan; b91401116@ntu.edu.tw (Y.-Y.L.); ycyeh2@vghtpe.gov.tw (Y.-C.Y.); hlho5@vghtpe.gov.tw (H.-L.H.); jennifer620jennifer620@gmail.com (H.-C.M.); 2 Cancer Progression Research Center, National Yang Ming Chiao Tung University, Taipei 112304, Taiwan; 3 Institute of Biomedical Informatics, National Yang Ming Chiao Tung University, Taipei 112304, Taiwan; yuchao@ym.edu.tw (Y.-C.W.); dustin3141@gmail.com (D.-W.Y.); candy1234234@gmail.com (C.-Y.H.); 4 Department of Biotechnology and Laboratory Science in Medicine, National Yang Ming Chiao Tung University, Taipei 112304, Taiwan; 5 Institute of Biochemistry and Molecular Biology, National Yang Ming Chiao Tung University, Taipei 112304, Taiwan; meychen@ym.edu.tw; 6 Department of Thoracic Surgery, Taipei Medical University Hospital, Taipei 110301, Taiwan; yuchungwu@tmu.edu.tw; 7 Institute of Clinical Medicine, National Yang Ming Chiao Tung University, Taipei 112304, Taiwan

**Keywords:** lung adenocarcinoma, brain metastasis, omics data analysis, *CDKN2A*, p16

## Abstract

Lung adenocarcinoma has a strong propensity to metastasize to the brain. The brain metastases are difficult to treat and can cause significant morbidity and mortality. Identifying patients with increased risk of developing brain metastasis can assist medical decision-making, facilitating a closer surveillance or justifying a preventive treatment. We analyzed 27 lung adenocarcinoma patients who received a primary lung tumor resection and developed metastases within 5 years after the surgery. Among these patients, 16 developed brain metastases and 11 developed non-brain metastases only. We performed targeted DNA sequencing, RNA sequencing and immunohistochemistry to characterize the difference between the primary tumors. We also compared our findings to the published data of brain-tropic and non-brain-tropic lung adenocarcinoma cell lines. The results demonstrated that the targeted tumor DNA sequencing did not reveal a significant difference between the groups, but the RNA sequencing identified 390 differentially expressed genes. A gene expression signature including *CDKN2A* could identify 100% of brain-metastasizing tumors with a 91% specificity. However, when compared to the differentially expressed genes between brain-tropic and non-brain-tropic lung cancer cell lines, a different set of genes was shared between the patient data and the cell line data, which include many genes implicated in the cancer-glia/neuron interaction. Our findings indicate that it is possible to identify lung adenocarcinoma patients at the highest risk for brain metastasis by analyzing the primary tumor. Further investigation is required to elucidate the mechanism behind these associations and to identify potential treatment targets.

## 1. Introduction

Lung cancer is the world-leading cause of cancer-related death [1], and lung adenocarcinoma has recently surpassed squamous cell carcinoma as the most common histology type [2]. Despite efforts in prevention, screening and treatment, many lung cancer patients still die of the disease, mostly because of distant metastasis. Among the metastatic sites, metastasis to the central nervous system, mainly the brain, is a major problem in patient care. Lung cancer, especially lung adenocarcinoma, has a strong propensity to metastasize to the brain. About 15% of patients already have brain metastasis at the time of the initial diagnosis [3]; more than 20% of all lung adenocarcinoma patients develop brain metastasis along their disease courses [4]. Of all cancer metastases to the brain, lung adenocarcinoma is the most common primary tumor, constituting 37% of all the cases [3]. The brain metastases can cause neurological deficits and increased intracranial pressure, resulting in significant morbidity and mortality. However, the current clinical practice has limited tools for the early detection and treatment of brain metastasis [5]. Because of the cost and radiation exposure related to brain imaging modalities, lung adenocarcinoma patients often do not receive regular brain imaging examinations until they develop symptoms and signs suspicious of brain metastasis. By this time point, multiple brain metastasis foci may have already developed, sometimes to a significant size, and surgical resection or stereotactic radiosurgery may not be feasible. Whole-brain irradiation and systemic therapy may be the patient’s only choices, but the irradiation may cause a significant cognitive function decline, and the chemotherapeutic agents and targeted therapies for driver mutations (such as tyrosine kinase inhibitors) invariably encounter the problem of tumor resistance. These treatments can control the brain metastasis temporarily at best, and most patients eventually die of disease progression.

One possible way to improve the management of lung adenocarcinoma-derived brain metastasis is to identify patients who are at the highest risk of developing brain metastasis. If such patients can be identified, implementing a regular brain imaging schedule may be justified, and the metastatic disease may be detected at an earlier time point to allow for a more effective treatment. A preventive treatment, either with irradiation or pharmaceutical agents, may also be considered for this selected group. To achieve this goal, several possible approaches may be taken. Many studies attempted to investigate the mechanism of lung adenocarcinoma brain metastasis by comparing the same patient’s primary lung tumor and a tumor from the brain metastatic site [6,7,8,9]. The rationale behind such an approach is that the “brain-tropic” clone of cells may be a minor clone in the primary tumor, which should be enriched in the brain site, and this phenomenon may allow us to identify genes and pathways important for this process. Indeed, studies by this method showed that *MYC*, *YAP1*, *MMP13* and other genes may contribute to the development of brain metastasis, and these may be potential treatment targets [6]. However, the information gained from this approach may not be useful for a risk stratification of patients before brain metastasis occurs, since detecting the minor clone in the primary tumor may be difficult. Another possibility is that some lung adenocarcinomas may have an inherently higher likelihood of metastasizing to the brain, either because of specific driver oncogenes or because of the tumor–host interaction. In this situation, the genotype or phenotype associated with the brain tropism should be present in both the entire primary tumor and the metastatic site, and a prediction of the brain metastasis by analyzing the primary tumor may be more feasible in this kind of situation. Indeed, studies have found genes that are altered in this manner [6], indicating that at least some brain metastases develop in this fashion. It is this group of patients that is the focus of our current study. We further hypothesized that, instead of comparing patients with brain metastasis to lung adenocarcinoma patients in general, comparing patients with brain metastasis to patients with non-brain metastasis may help us identify features specifically related to brain-tropism. Since both groups of patients have metastatic diseases, any difference remaining may be more likely related to the brain-metastasizing mechanisms.

In order to address the unmet clinical need and to test our hypothesis, we retrospectively analyzed lung adenocarcinoma patients who received a surgical primary tumor resection and later developed brain or non-brain metastasis within 5 years in a single medical center. We first performed a targeted next-generation sequencing of the tumors to investigate their genetic composition. We also performed a comprehensive transcriptome analysis of the primary tumor tissue by RNA sequencing (RNA-seq) to identify differentially expressed genes (DE genes) between the two groups. Based on the difference between the groups, we proposed algorithms to segregate lung adenocarcinoma patients into the high risk/low risk categories for brain metastasis. We further compared our patient study results with the difference found in the study of brain-tropic and non-brain-tropic lung adenocarcinoma cell lines in animal models to look for common mechanisms between the two systems.

## 2. Results

### 2.1. Basic Clinical and Pathological Characteristics

The basic characteristics of the patients are summarized in Table 1. A total of 16 patients who developed brain metastasis within 5 years after a surgical resection of the primary lung adenocarcinoma were identified, while 11 patients developed only non-brain metastasis in the same time window. These two groups of patients had similar age, size of primary tumor and experience of adjuvant chemotherapy. Of notice, a larger proportion of patients with brain metastasis were female (male to female ratio = 6:10), while more patients with non-brain metastasis were male than female (male to female ratio = 8:3). On the contrary, fewer patients with brain metastasis had a smoking history compared to those with non-brain metastasis (43.8 % vs. 63.6%).

About the pathological features of their diseases, the predominant growth pattern in the primary tumors was mostly acinar in both groups. Regarding the growth patterns traditionally considered of high risk for metastasis (micropapillary and solid), 50% of the brain-metastasizing tumors contained predominantly either one of these two patterns, compared to 45.5% of the non-brain metastasizing tumors, although micropapillary-predominance was more common in the brain-metastasizing group. The distribution of the T stage and the N stage at the time of surgery was similar between the two groups, except that the brain-metastasizing group had more N2 cases (31.3% vs. 27.3%). Overall, some difference was observed in sex ratio, smoking history, frequency of histological micropapillary predominance and N2 stage, but none of these differences was of sufficient magnitude to allow for its use as clinical guidance for brain metastasis risk stratification, and the differences were all statistically non-significant (*p* > 0.05). The actual timeline of the brain/non-brain-metastasis occurrence and the follow-up length for each individual case are shown in Figure S1.

### 2.2. No significant Genomic Difference Was Identified between Brain-Metastasizing and Non-Brain-Metastasizing Lung Adenocarcinomas by Targeted Next-Generation Sequencing

**Figure 1:**
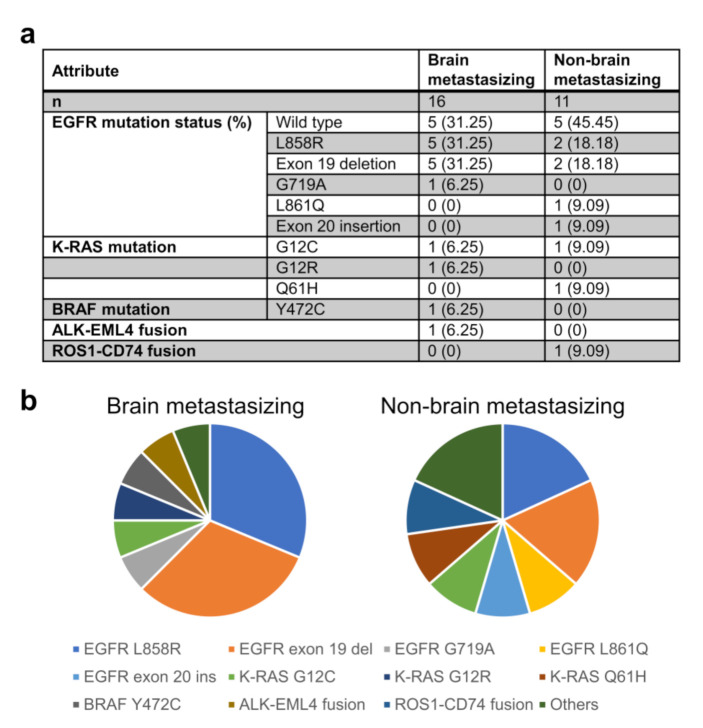
Presence of common lung adenocarcinoma driver mutations and gene fusions in the patient cohort. Panel (**a**) lists the number and percentage of patient tumors carrying each common driver mutations and gene fusions. Panel (**b**) shows the distribution of the above-mentioned genetic alterations in pie chart format.

We compared the genomic composition of the primary lung tumors of the two groups of patients with the FoundationOne CDx targeted DNA sequencing panel (Foundation Medicine, Cambridge, MA, USA) (Figure 1). In our patient population, we found that a *EGFR* gene alteration was present in 68.75% of the patients with brain metastasis and 54.55% of those with non-brain metastasis. Among those with the *EGFR* alteration, the two most common alterations were equally found in both groups (five cases each for L858R mutation and exon 19 deletion in the brain metastasis group; two cases each in the non-brain metastasis group). The other, less common *EGFR* alterations were observed in single patients. In summary, there is no significant correlation between the *EGFR* gene alteration and brain metastasis (Fisher’s exact test, *p* = 0.49). Chromosome rearrangements involving *ALK* and *ROS1* were found in only one patient in the brain metastasis group (*ALK-EML4*) and one in the non-brain metastasis group (*CD74-ROS1*). Variants of *K-RAS* and *BRAF* mutations also occurred in single patients in each group. We did not find any other single genomic alteration that was significantly different between the two groups; other than the *EGFR* alterations mentioned above, no other genetic alteration was found in more than three cases (Table S1). None of the sequenced cases showed microsatellite instability (MSI). As for the tumor mutation burden, the average mutations per megabase were 4.59 in the brain-metastasizing group and 5.30 in the non-brain-metastasizing group; the difference was not significant using the Wilcoxon rank sum test (*p* = 0.7221).

### 2.3. The mRNA Expression Profile, including CDKN2A, Is Significantly Different between Brain-Metastasizing and Non-Brain-Metastasizing Lung Adenocarcinomas

**Figure 2:**
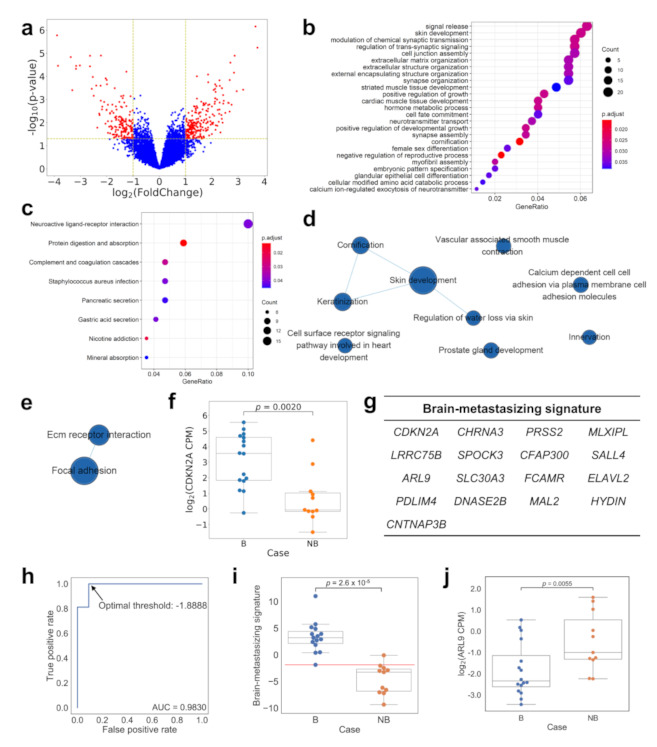
Comparing the gene expression profile of brain-metastasizing and non-brain-metastasizing lung adenocarcinomas using RNA-seq. The Volcano plot (panel (**a**)) showed differentially expressed genes (DE genes) with at least two-fold expression difference and *p* < 0.05 between the two groups by DESeq2. A total of 390 genes were identified. The GO enrichment analysis (panel (**b**)) and the KEGG pathway enrichment analysis (panel (**c**)) of the DE genes highlighted multiple groups of genes and pathways, notably the cellular interaction with extracellular matrix. The visualization of enriched GO terms or KEGG pathways were presented with clusterProfiler [10], and only the top 10 enriched GO terms were shown. The GSEA with GO (panel (**d**)) and KEGG (panel (**e**)) also found an enrichment of several similar gene sets, which were visualized by EnrichmentMap [11]. However, when the ability of the individual DE gene to segregate the two groups of tumors was analyzed, the top gene with the greatest AUC value in the ROC analysis was *CDKN2A*. The dot plot (panel (**f**)) of *CDKN2A* expression showed that while brain-metastasizing tumors have a range of expression levels, most non-brain-metastasizing tumors express very little of this gene (*p* = 0.0020, Mann–Whitney U test). A 17-gene brain-metastasizing signature (panel (**g**)) was identified for classification. The optimal threshold was determined as −1.89, as indicated in the ROC curve (panel (**h**)). The dot plot (panel (**i**)) showed that the brain-metastasizing signature was significantly higher in the brain-metastasizing group (*p* = 2.6 × 10^−5^, Mann–Whitney U test). The red line indicated the optimal threshold for classification. The dot plot (panel (**j**)) of *ARL9* expression showed that the expression was significantly lower in brain-metastasizing tumors (*p* = 0.0055, Mann–Whitney U test). B: brain-metastasizing, NB: non-brain-metastasizing.

We next compared the transcriptome of the two groups of primary tumors via a RNA-seq of fresh-frozen tumor tissue (Figure 2). A volcano plot (Figure 2a) showed the differentially expressed genes (DE genes) with an at least two-fold expression difference and a *p* value less than 0.05, as determined by the DESeq2 program. A total of 390 DE genes were identified. A Gene Ontology (GO) enrichment analysis (Figure 2b, Table S2) showed multiple biological processes varying between the two groups of tumors, notably including an “extracellular matrix organization”, which may be related to their metastasis behavior. Interestingly, biological processes related to the nervous system, such as synaptic transmission and assembly, are also highlighted by the analysis, while a Kyoto Encyclopedia of Genes and Genomes (KEGG) enrichment analysis also showed that neuroactive ligand-receptor interaction-related genes are differentially expressed between the groups (Figure 2c). The Gene Set Enrichment Analysis (GSEA) based on GO (Figure 2d, Table S2) and KEGG (Figure 2e, Table S2) also pointed out that genes related to cell adhesion and the extracellular matrix were differentially expressed. When Receiver Operating Characteristic (ROC) curves were used to analyze the ability of individual genes to correctly segregate cases into brain-metastasizing and non-brain-metastasizing, the gene with top performance was *CDKN2A*, with an area under curve (AUC) of 0.86. Using the expression of this single gene in the primary tumor could correctly segregate cases into brain-metastasizing and non-brain metastasizing with a sensitivity of 93.8%, a specificity of 81.8%, a positive predictive value (PPV) of 88.2% and a negative predictive value (NPV) of 90% (Table S3). A dot plot (Figure 2f) showed that the brain metastasizing tumors demonstrated a range of *CDKN2A* expression, while most of the non-brain-metastasizing tumors showed a low *CDKN2A* expression. The difference was statistically significant (*p* = 0.002). Based on the gene list ranked with AUC, a stepwise method was used to build a 17-gene brain-metastasizing signature (Figure 2g, Table S3). With the optimal threshold −1.89 determined by the ROC curve (Figure 2h), the brain-metastasizing signature was shown to identify 100% of brain-metastasizing tumors with a 91% specificity (Figure 2i). A leave-one-out cross validation was further applied, demonstrating that the signature had a 60% precision and a 75% recall. In addition, the expression of *ARL9* was significantly lower in brain-metastasizing tumors than in non-brain-metastasizing tumors. (Figure 2j). The significance of this gene will be explained later in the article.

**Figure 3:**
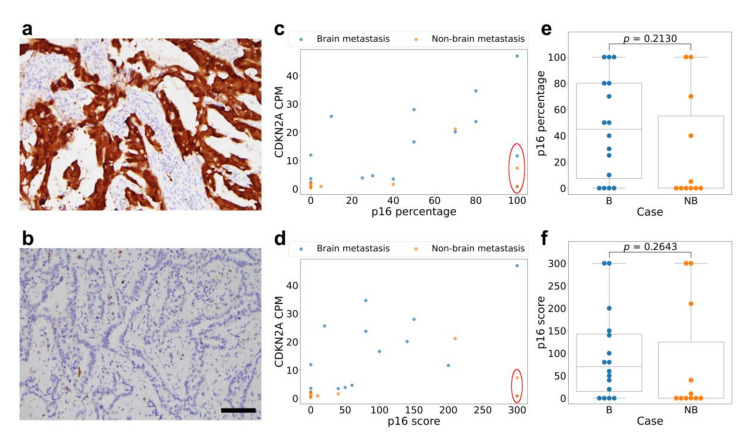
The p16 immunohistochemical staining of lung adenocarcinoma tissue shows a moderate correlation with the *CDKN2A* RNA expression. Representative photographs show one tumor with 100% strong-intensity (3+) p16 staining (panel (**a**)) compared to another tumor with 0% (negative, 0 intensity) staining (panel (**b**)). The percentage of tumor cells positive for p16 shows a moderate correlation with the *CDKN2A* RNA expression level (panel (**c**)), but the correlation is not significant for the p16 staining H-score (panel (**d**)). Note that 4 cases deviating from the correlation form a group and share the feature of low *CDKN2A* RNA expression and high p16 positive percentage and score (red circle). Of these cases, 2 belong to the brain metastasizing group and 2 belong to the non-brain-metastasizing group. Box plots of p16-positive percentage (panel (**e**)) and p16 H-score (panel (**f**)) show that the brain-metastasizing cases tend to have a variable staining of p16, some reaching high levels, while non-brain-metastasizing cases tend to have low p16 staining. However, the difference was not clear-cut nor statistically significant (*p* = 0.21 for the percentage and 0.26 for the H-score, Mann–Whitney U test). Scale bar: 100 micrometer. B: brain-metastasizing, NB: non-brain-metastasizing.

To assess the RNA expression difference at the protein level, we performed immunohistochemistry (IHC) for p16, the protein product of the *CDKN2A* gene, on a tissue microarray constructed from the patients’ archived formalin-fixed, paraffin-embedded (FFPE) lung tumor tissue. (Figure 3a,b). We specifically chose this target because among the protein products of the genes in our list of high AUC candidates, p16 immunohistochemistry is the most widely performed in pathology laboratories. However, the correlation between the tumor *CDKN2A* mRNA expression level, p16-positive cell percentage and p16 immunohistochemistry H-score was only moderate (Figure 3c,d). The Pearson correlation coefficient between the p16-positive cell percentage and the *CDKN2A* expression was 0.47 (*p* = 0.014), while the correlation coefficient between the p16 immunohistochemistry H-score and the *CDKN2A* expression was 0.32 (*p* = 0.099). We noticed a few cases with very diffuse (100%) and strong p16 immunostaining but low mRNA expression (CPM < 12 in RNA-seq). These include two cases in the brain-metastasizing group and two cases in the non-brain-metastasizing group. Other than these cases, we found that the rest of brain-metastasizing tumors are more frequently positive for p16 staining with variable positive percentages and intensity, while the non-brain-metastasizing tumors show limited or no p16 staining. Nevertheless, the overall p16 staining was not significantly different between the two groups, either looking at the p16-positive cell percentage or the H-score (*p* = 0.21 and 0.26, respectively) (Figure 3e,f).

### 2.4. Comparing the Gene Expression Pattern between Brain-Metastasizing Patient Tumors and Brain-Tropic Lung Adenocarcinoma Cell Lines Showed a Small Set of Shared Differentially Expressed Genes

**Figure 4:**
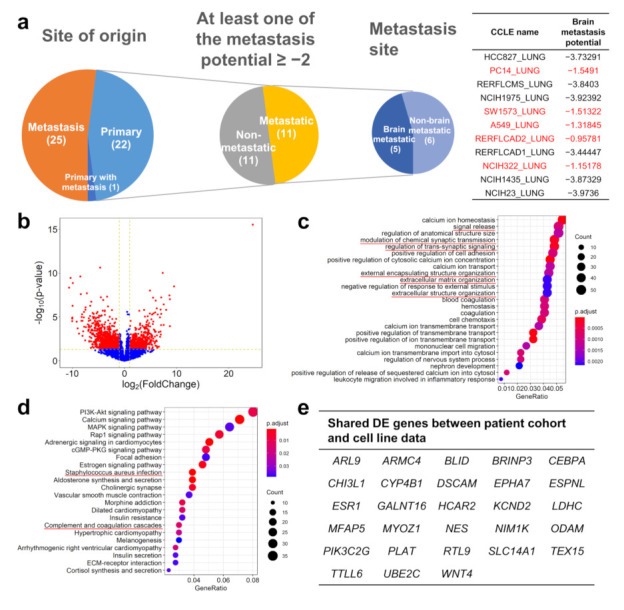
Analysis of brain-tropic and non-brain-tropic lung adenocarcinoma cell lines identified by the MetMap project showed differentially altered pathways and genes in common with lung cancer patient data. (**a**) Among the 48 lung adenocarcinoma cell lines analyzed by the MetMap project, 22 were from primary tumors, and among them 11 were found to have substantial metastatic potential. Five of these 11 were found to have a higher brain metastasis potential, while 6 were considered to have a low brain metastasis potential. (**b**) Analysis of cell line RNA-seq data from the CCLE database showed that the brain-tropic and non-brain-tropic cell lines have 1079 differentially expressed genes with an at least 2-fold expression difference and a *p* value lower than 0.05. The GO enrichment analysis (**c**) and the KEGG pathway enrichment analysis (**d**) showed multiple differences between the two groups of cell lines; the representative GO terms or KEGG pathways that were also identified in our patient cohort analysis were highlighted with red color. (**e**) Twenty-eight genes were found to be differentially expressed in the same direction in both the cell line analysis and the patient cohort analysis.

We hypothesized that lung adenocarcinoma cell lines with a higher propensity to metastasize to the brain may share common gene expression features with the lung adenocarcinoma patients’ lung tumors that produced brain metastases. We examined the recently published MetMap [12] database to look for lung adenocarcinoma cell lines with a different metastasis tropism. In this database, various cell lines were genetically barcoded and intracardiac-injected into immunodeficient mice, then traced in different organs using single-cell sequencing technology. Among the tested cell lines, there were 11 derived from primary lung adenocarcinoma tumors with metastasis potential, and five of them were determined to have higher brain metastasis potential (Figure 4a). We retrieved the gene expression profile of these 11 cell lines from the Cancer Cell Line Encyclopedia (CCLE) database [13] and compared those with higher brain metastasis potential to those with lower potential. We found 1079 genes differentially expressed between the two groups (Figure 4b). The GO enrichment and KEGG pathway enrichment analysis results are shown in Figure 4c,d. Interestingly, we found that multiple biological processes highlighted the overlap with those found in our patient cohort analysis. In the GO enrichment analysis, “signal release”, “modulation of chemical synaptic transmission”, “regulation of trans-synaptic signaling”, “extracellular matrix organization”, “extracellular structure organization” and “extracellular encapsulating structure organization” were also enriched in our patient cohort analysis and appear to be related to the nervous system or cell adhesion. The overlapping results in the KEGG pathway analysis include “complement and coagulation cascades” and “Staphylococcus aureus infection”, which may also contribute to brain metastasis (see Discussion below). We further compared individual genes on the cell line DE gene list with the DE gene list derived from our patient cohort. We found 28 genes that were differentially expressed both between the brain-tropic/non-brain-tropic cell lines and between the brain-metastasizing/non-brain-metastasizing patient tumors, and with the difference in the same direction (e.g., higher in the brain-tropic cell lines and higher in the brain-metastasizing patient tumors) (Figure 4e). Noticeably, only one gene in the patient cohort-derived brain-metastasizing signature, *ARL9*, was included in this 28-gene set (Figure 2j). In fact, the expression of classical immune-related genes, such as CD3 (hallmark of T lymphocytes) and CD20 (hallmark of B lymphocytes), are detected in our patient cohort (average CPM: CD20 14.99, CD3D 35.59, CD3E 30.58, CD3G 15.40) but not detected in the cell line experiment (average CPM: CD20 0.16, CD3D 0.04, CD3E 0.07, CD3G 0.01), highlighting the absence of the role of the immune system in the cell line experiment. This reflects the fundamental difference between patient tumors and cancer cell line behavior in animal models, yet those 28 differentially expressed genes shared between these two very different systems may warrant further study because they may be related to fundamental principles of lung cancer brain metastasis.

## 3. Discussion

We proposed an algorithm to stratify lung adenocarcinoma patients into those with high risk for brain metastasis development and those with low risk, potentially useful for guiding the clinical management of patients receiving curative primary lung tumor resection. If the algorithm can be verified in a larger, statistically powered cohort in a prospective study, at the detection of the first metastasis, if not in the brain, the patient’s primary tumor may be analyzed according to our algorithm, and the patient’s brain metastasis risk assessed. If the risk is high, then the patient may begin to receive regular brain imaging even without neurological signs and symptoms, for the purpose of early detection. Preventive treatment may also be considered, although the risk and benefit of such treatments may require further studies to confirm. For neurologically asymptomatic patients who received brain imaging either during re-staging, because of a non-brain metastasis, or for surveillance only, sometimes small, equivocal lesions will be detected. Our algorithm may also provide the clinician and patient with more risk-stratification information in terms of how to manage such image findings. In a broader sense, any lung adenocarcinoma patient with distant metastasis may be analyzed for their risk of brain metastasis. However, whether our findings still hold true in this population may require further confirmation, and it is of interest to know if needle biopsies of the primary tumor or even a non-brain metastatic site can be used for this purpose.

Among the genes included in our prediction model, *CDKN2A* is most well-known for its role in tumor development. However, unlike the previous report that showed *CDKN2A* mutation was associated with brain metastasis [6], we found its over-expression is. Although many previous studies have characterized the phenomenon of *CDKN2A*/p16 loss in lung adenocarcinoma and its relationship with a poor prognosis [14,15,16], many studies also reported that *CDKN2A*/p16 expression is not related to the prognosis [17,18,19], or even that an over-expression is related to a poor prognosis [20]. Indeed, the role of *CDKN2A*/p16 in the formation of brain metastasis by lung adenocarcinoma has rarely been specifically studied. One report showed that the metastatic adenocarcinoma cells from the brain site express more p16 than the primary lung tumor [21]. To our knowledge, our study is the first to demonstrate a relationship between *CDKN2A* expression and the brain tropism of metastasis. The difference between our findings and the previous report [6] may be attributed to the different patient population studied; in our cohort, a high proportion (63%) of patients have EGFR gene alterations, which is common in east Asian lung adenocarcinoma patients in general but uncommon in Western countries. As for the mechanism whereby *CDKN2A* expression contributes to brain metastasis, it is conspicuous that traditional genes and pathways related to the *CDKN2A* function, i.e., cell-cycle-related genes and pathways, are not significantly differentially expressed between brain-metastasizing and non-brain-metastasizing tumors in this study. A possible explanation is that the *CDKN2A* expression difference may indicate a compensatory mechanism to various cell cycle dysregulations (e.g., responding to *RB* loss or *CDK4*/*CDK6* gene amplification), and its function in brain metastasis lies in non-cell-cycle regulatory roles. One study on head and neck squamous cell carcinoma showed that p16 expression can stimulate lymphangiogenesis but inhibit angiogenesis, which may correlate with the strong tendency of p16-positive head and neck squamous cell carcinoma to spread through the lymphatic system [22]. However, such a mechanism cannot explain the brain metastasis behavior of lung adenocarcinoma, which most likely occurs via the hematogenous route. In a mouse non-small-cell lung-cancer model, the inhibition of CDK4/6, the downstream target of p16, resulted in increased CD4 and CD8 T cell infiltration in the tumor [23]. It is now known that adaptive immune cells influence tumor angiogenesis and metastasis behavior [24]. Inflammation-associated angiogenesis may contribute to the establishment of metastasis specifically in the brain’s microenvironment, which is reported to be the most inefficient and therefore crucial step in brain metastasis establishment [25]. Further studies are required to elucidate the mechanism behind the association we discovered.

The regulation of *CDKN2A*/p16 expression in cancer cells is complex [26]. Its loss is often ascribed to the deletion of the gene or the methylation of its promoter, but its over-expression is less understood. The cellular response to stress or other oncogenic environmental factors may drive its expression, and its normal function of inhibiting cell proliferation is negated by other mechanisms. In lung cancer, smoking has been linked to p16 over-expression [27]. Some studies reported the detection of human papilloma virus, a known cause of p16 over-expression, in lung cancer [28,29,30], while others did not [31,32]. In addition, we also noted in our study a group of patients with a low *CDKN2A* RNA level but high p16 immunohistochemistry staining. The post-translational regulation of p16 is not very well understood. The protein is generally considered short-lived and rapidly degraded by the proteasome in minutes to hours [26]. The interaction between p16 and proteasome activator REGγ has been shown to be required for its degradation [33]. Whether such interactions were disrupted in our cases with discrepant *CDKN2A* RNA-p16 protein levels requires further investigation. Another pathway of p16 degradation is through autophagy [34]. We found that in three of the four cases with low *CDKN2A* mRNA expression but strong p16 protein staining, the tumor harbors either *PIK3CA* mutation, *PIK3CB* amplification or loss of *PTEN* gene (Table S2, case B6, NB7, NB8). These genomic alterations can potentially increase the activity of the PI3K signal transduction pathway, which is known to be able to suppress autophagy [35]. *PIK3CA*, *PIK3CB* or *PTEN* alteration was not observed in cases without the *CDKN2A*/p16 discrepancy. The correlation between the PI3K pathway, autophagy and p16 requires further study to clarify.

The analysis of brain-tropic vs. non-brain-tropic lung adenocarcinoma cell lines based on their behavior in immunodeficient mice demonstrated a different gene expression pattern between the two groups, yet not many of these differentially expressed genes were found in our analysis of patient tumors. We think this is because the patient tumors and the cell line/mouse model systems have many important differences, notably the absence of immune surveillance in the cell line/mouse model. A significant limitation of our study is the relatively small number of patients studied, and a lack of testing cohort to verify the brain metastasis-related gene expression signature we identified, a role that the comparison with the cancer cell line data can only partially fill. However, despite these differences, we still identified 28 genes that were differentially expressed in the same manner in both systems, many of which were related to neurological processes. The GO enrichment analysis also found that genes related to synaptic transmission and signaling were enriched among the differentially expressed genes in both the patient cohort data and the cell line data. It is known that cancer cells can interact with cells in the central nervous system, such as neurons and glia cells, to facilitate the establishment of brain metastasis [29]. One gene, *DSCAM*, is more highly expressed in both the brain-metastasizing patient tumors and brain-tropic cell lines in our analyses. This gene encodes a cell adhesion molecule involved in glutamate synapse formation [36]. It has been reported in breast cancer that cancer cells can mimic the reciprocal relationship between astrocytes and neurons, metabolize glutamate to GABA and promote tumor cell proliferation [37]. On the contrary, our analysis found that both the brain-metastasizing patient tumors and brain-tropic cell lines express less mRNA of *PLAT* than their non-brain-metastasizing/tropic counterparts. *PLAT* encodes a tissue type plasminogen activator, and it has been shown that its activation target, plasmin, can inhibit brain metastasis by releasing FasL from astrocytes to promote cancer cell death, as well as inactivating the adhesion molecule L1CAM important for cancer spreading [38]. These findings demonstrate that cancer-glia/neuron interaction may play a fundamental role in lung cancer brain metastasis development, which transcends different species such as mouse and man.

In summary, it is possible to identify lung adenocarcinoma patients with a high risk of brain metastasis by analyzing the primary tumor. Our current study is limited by its relatively small sample size and its retrospective nature. Our RNA analysis was performed with fresh frozen tissue obtained during primary tumor surgery. Whether archived tissue can generate similar results is not known. A prospective study with larger patient numbers using FFPE tissue is required to validate these findings and to prove their clinical utility. An animal experiment comparing brain-tropic and non-brain-tropic metastatic lung adenocarcinoma in an immune-competent environment using genetically engineered models [39] is also required to validate our findings and further dissect the biological mechanisms. Therapies targeting the p16/CDK/Rb pathway may be evaluated for its role in the prevention or treatment of brain metastasis.

## 4. Materials and Methods

### 4.1. Patient Selection

We retrospectively enrolled patients who were at least 20 years old and received surgery for lung adenocarcinoma at Taipei Veterans General Hospital from 2007 to 2012. The inclusion and exclusion criteria are: (1) The patient received a primary lung tumor resection during this period, either by lobectomy or wedge resection. During surgery, the tumor was judged by the surgeon to be of sufficient size to allow the direct freezing of a portion of tumor specimen in liquid nitrogen. (2) The pathological diagnosis of the primary lung tumor was a pure adenocarcinoma of lung origin, with no squamous component, small cell component, mucinous phenotype or other special histology types. (3) The patient did not have another malignancy diagnosed from 5 years before to 5 years after the lung tumor resection date. (4) The patient did not receive neoadjuvant therapy before surgery (adjuvant therapy was allowed). (5) The patient had clinically or pathologically documented distant metastasis detected within 5 years after the surgery. Patients with only lung-to-lung metastasis were excluded because of the possible confounding factor of multiple primary lung carcinoma. Similarly, patients with multiple lung tumors at the time of surgery, in whom the primary tumor cannot be clearly determined by a clinical or pathological examination, were also excluded. Patients with only pleural metastasis were also excluded, considering the possible route difference (direct seeding versus hematogenous spreading) between pleural metastasis and other distal organ metastasis. (6) Follow up period: patients who developed brain metastasis within 5 years were all included, regardless of whether they had metastasis to another organ. Those who developed only non-brain metastasis were included only if the patients had at least 2.5 years of clinical follow-up after the surgery, or if the patient died within 5 years. This study was approved by the Institutional Review Board (IRB) of Taipei Veterans General Hospital (ID No. 2016-09-031AC) in accordance with the Declaration of Helsinki. The informed consent requirement was waived.

### 4.2. Targeted DNA Next-Generation Sequencing to Detect Genomic Alterations

Formalin-fixed, paraffin-embedded primary lung tumor sections from the patients were sent to Foundation Medicine (MA, USA) for targeted DNA sequencing using the FoundationOne CDx panel, which includes 324 known cancer-related genes for substitution, insertion/deletion, copy number variations and rearrangements. Microsatellite stability and tumor mutation burden were also assessed. The sample preparation and analysis process were performed according to the Foundation Medicine protocol.

### 4.3. Transcriptome Analysis and Identification of Differentially Expressed Genes

Total RNA was extracted from the lung tumor tissue fragments (approximately 0.5 × 0.5 × 0.5 cm) preserved in liquid nitrogen at the time of the surgery. The extraction was performed using a QIAGEN RNeasy Mini Kit (QIAGEN, Germantown, MD, USA). The cDNA library was built from the RNA with Illumina TruSeq RNA Exome Kit (Illumina, San Diego, CA, USA). 150bp paired-end sequencing, 50 million reads per sample, was performed on the Illumina HiSeq 4000 platform.

The raw sequencing data were aligned to the reference human genome (GRch38) using the STAR software (version 2.7.2a) [40]. The reads mapped to each gene were enumerated using HTSeq (version 0.11.1) [41]. After low-count filtering by edgeR [42], the read counts of protein-coding genes were fed into DESeq2 [43] to determine the differentially expressed genes between brain-metastasizing and non-brain-metastasizing tumors. Meanwhile, the CPM (counts per million) or log_2_CPM value was calculated for each DE gene. The list of DE genes was subjected to a GO (Gene Ontology) enrichment analysis a and KEGG (Kyoto Encyclopedia of Genes and Genomes) pathway enrichment analysis by clusterProfiler [10]. A Gene Set Enrichment Analysis (GSEA) [44] was also applied to investigate the enriched function/pathways. The receiver operating characteristic (ROC) curve of each individual DE gene for its ability to segregate cases into brain-metastasizing and non-brain-metastasizing was plotted, and DE genes with top area under curve (AUC) values were identified. Additionally, a stepwise selection method based on a principal component analysis (PCA) was proposed to identify the optimal gene set for classifying brain-metastasizing samples. Specifically, according to the gene list with a ranked AUC value, one gene with a top AUC value was added into the gene set in each round. Then, PCA was applied using the expression profiles of the gene set. Consequently, the value of the first principal component for each sample was used for classification and the corresponding AUC was calculated for the specific gene set. This gene-adding process continued until the AUC could not be increased in the next five rounds. In this way, the gene set with the highest AUC was defined as the brain-metastasizing signature for classification. A leave-one-out cross validation was further employed to test the classification performance.

### 4.4. Immunohistochemistry

We examined the differentially expressed genes and identified genes of particular interest, i.e., genes with a top AUC value in the ROC plots, and for which there are antibodies commercially available against their protein products. We chose *CDKN2A* (p16, clone E6H4, Ventana Medical Systems, Oro Valley, AZ, USA) as our target. Immunohistochemistry was performed to corroborate the RNA expression differences on tissue microarrays.

Tissue microarrays were constructed from archived formalin-fixed, paraffin-embedded (FFPE) lung tumor tissue from the patients. All specimens were fixed for 6–72 h before embedding in paraffin. Two cores, each with a diameter of 2 mm, were taken from representative tumor areas of each patient. Four micrometer-thick sections were cut from the arrays and attached onto slides. One section was stained with hematoxylin and eosin for morphology evaluation. The other section was stained with the primary antibody on the Leica Bond-Max (Leica Biosystems, Mount Waverley, VIC, Australia) automated staining platform. The slides were stained with a primary antibody at room temperature for 15 min and then treated with the Bond Polymer Refine Detection Kit (Leica Microsystems, Milton Keynes, UK). The sections were counter-stained with hematoxylin. The percentage of tumor cells positive for p16 was recorded, and the immunohistochemistry H-score was calculated.

### 4.5. Comparison of Gene Expression Profile between Brain-Tropic Lung Adenocarcinoma Cell Lines and Patients with Brain-Metastasizing Lung Adenocarcinoma

A recently published database (MetMap) [12] described the metastasis organ tropism of various human cancer cell lines in an immunodeficient mouse model based on single-cell sequencing technology. In this database, 11 human lung adenocarcinoma cell lines derived from primary tumors with metastatic potential were identified. These cell lines were separated into brain-tropic versus non-brain-tropic based on their brain metastasis potential determined by the MetMap project. A potential greater than −2 (on a log_10_ scale) is considered brain-tropic, and a value less than −2 is considered non-brain tropic. The RNA-seq-based gene expression profile of these cell lines was retrieved from the Cancer Cell Line Encyclopedia (CCLE) database [13]. Differentially expressed genes between the brain-tropic and non-brain-tropic cell lines were determined with DEseq2, similarly to the analysis performed on our lung cancer patient specimen RNA-seq data. A GO enrichment analysis and a KEGG pathway enrichment analysis were also performed for the identified DE genes. We compared the DE genes from the MetMap/CCLE cell line data to our patient tumor data and identified the overlapping DE genes with the same direction of difference (e.g., higher in both the brain-tropic cell line and the brain-metastasizing patient tumor).

### 4.6. Statistical Analysis

In general, a Student’s t test was performed for the continuous variables, and a Chi-squared test or Fisher’s exact test was performed for the categorical variables to determine whether there was a significant difference between the brain-metastasizing and non-brain-metastasizing groups. A Mann–Whitney U test was performed to compare the *CDKN2A* RNA expression level and p16 immunohistochemistry staining between the two groups. A Pearson correlation coefficient was calculated to demonstrate the correlation between the *CDKN2A* mRNA expression and p16 immunohistochemistry results. A Wilcoxon rank sum test was performed to compare the tumor mutation burden between the two groups. A *p* value less than 0.05 was considered significant.

## Figures and Tables

**Table 1:** Basic clinical and pathological information of patients.

Attribute		Brain Metastasizing	Non-Brain Metastasizing	*p* Value
*n*		16	11	
Mean age (range)		62 (45–78)	67 (46–77)	0.19
Male sex (%)		6 (37.5)	8 (72.7)	0.12
Smoking history (%)		7 (43.8)	7 (63.6)	0.44
Mean tumor size (S.D.)		2.9 (1.1)	3.4 (1.9)	0.40
Received adjuvant chemotherapy (%)		11 (68.8)	8 (72.7)	1
Predominant growth pattern in primary tumor	Acinar (%)	7 (43.7)	5 (45.4)	0.55
	Papillary (%)	1 (6.3)	1 (9.1)	
	Micropapillary (%)	5 (31.3)	1 (9.1)	
	Solid (%)	3 (18.7)	4 (36.4)	
T stage (%)	T1a	1 (6.3)	1 (9.1)	0.28
	T1b	3 (18.7)	3 (27.3)	
	T2a	11 (68.7)	4 (36.3)	
	T2b	1 (6.3)	1 (9.1)	
	T3	0 (0)	2 (18.2)	
N stage (%)	N0	8 (50.0)	5 (45.5)	1
	N1	3 (18.7)	3 (27.3)	
	N2	5 (31.3)	3 (27.3)	

S.D.: standard deviation. T stage was reported according to AJCC 7th Ed.

## Data Availability

The RNA-seq data obtained in the current study were deposited in the NCBI Sequence Read Archive (SRA) under the accession number PRJNA649988. The FoundationOne CDx panel DNA sequencing data that support the findings of this study are available from Foundation Medicine (MA, USA), but restrictions apply to the availability of these data, which were used under license for the current study, and so are not publicly available. However, the data are available from the authors upon reasonable request and with the permission of Foundation Medicine.

## Supplementary Materials

The following are available online at https://www.mdpi.com/article/10.3390/ijms222413374/s1.
