# Supplementary figures and images for: Gene Expression Profile in Primary Tumor Is Associated with Brain-Tropism of Metastasis from Lung Adenocarcinoma
Source: Int J Mol Sci. 2021 Dec 13;22(24):13374. doi: 10.3390/ijms222413374 (PMC8703941; doi:10.3390/ijms222413374)

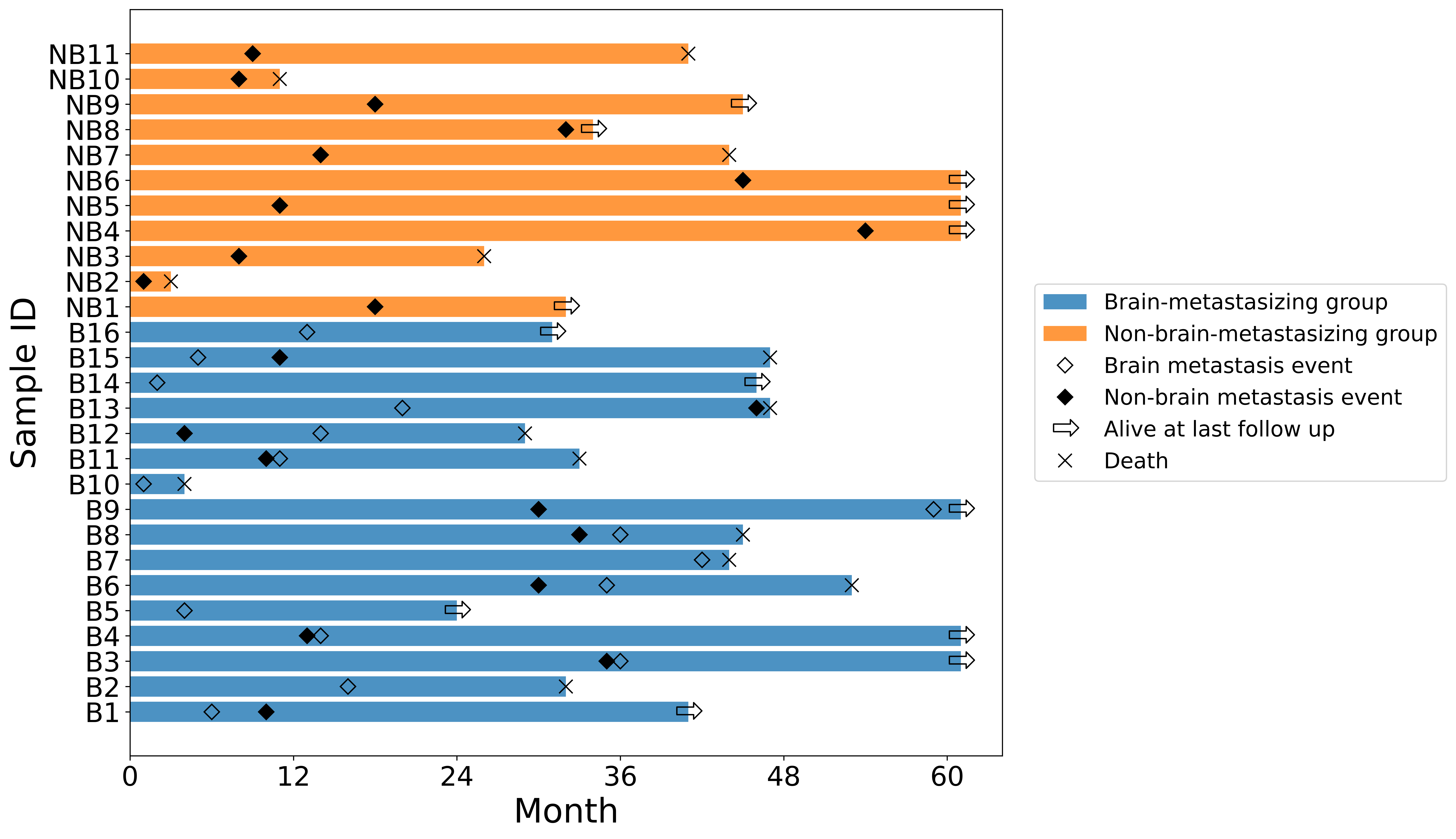

Supplement: Supplementary file 1 [file ijms-22-13374-s001.zip › Supplementary Figure 1_Swimmer plot.tiff]
